# Reconstruction and Analysis of Transcription Factor–miRNA Co-Regulatory Feed-Forward Loops in Human Cancers Using Filter-Wrapper Feature Selection

**DOI:** 10.1371/journal.pone.0078197

**Published:** 2013-10-29

**Authors:** Chen Peng, Minghui Wang, Yi Shen, Huanqing Feng, Ao Li

**Affiliations:** 1 School of Information Science and Technology, University of Science and Technology of China, Hefei, Anhui, China; 2 Research Centers for Biomedical Engineering, University of Science and Technology of China, Hefei, Anhui, China; Memorial Sloan Kettering Cancer Center, United States of America

## Abstract

**Background:**

As one of the most common types of co-regulatory motifs, feed-forward loops (FFLs) control many cell functions and play an important role in human cancers. Therefore, it is crucial to reconstruct and analyze cancer-related FFLs that are controlled by transcription factor (TF) and microRNA (miRNA) simultaneously, in order to find out how miRNAs and TFs cooperate with each other in cancer cells and how they contribute to carcinogenesis. Current FFL studies rely on predicted regulation information and therefore suffer the false positive issue in prediction results. More critically, FFLs generated by existing approaches cannot represent the dynamic and conditional regulation relationship under different experimental conditions.

**Methodology/Principal Findings:**

In this study, we proposed a novel filter-wrapper feature selection method to accurately identify co-regulatory mechanism by incorporating prior information from predicted regulatory interactions with parallel miRNA/mRNA expression datasets. By applying this method, we reconstructed 208 and 110 TF-miRNA co-regulatory FFLs from human pan-cancer and prostate datasets, respectively. Further analysis of these cancer-related FFLs showed that the top-ranking TF STAT3 and miRNA hsa-let-7e are key regulators implicated in human cancers, which have regulated targets significantly enriched in cellular process regulations and signaling pathways that are involved in carcinogenesis.

**Conclusions/Significance:**

In this study, we introduced an efficient computational approach to reconstruct co-regulatory FFLs by accurately identifying gene co-regulatory interactions. The strength of the proposed feature selection method lies in the fact it can precisely filter out false positives in predicted regulatory interactions by quantitatively modeling the complex co-regulation of target genes mediated by TFs and miRNAs simultaneously. Moreover, the proposed feature selection method can be generally applied to other gene regulation studies using parallel expression data with respect to different biological contexts.

## Introduction

Central to all biological organisms, the deciphering of complicated gene regulations between a group of regulators and target genes is crucial to learn the intracellular physiological activities and functions in the molecular level. It also helps to understand the internal mechanisms of complex diseases *in vivo*. The regulators in gene regulations include transcription factors (TFs), which are proteins binding to specific sites in the promoter regions of target genes, thus activating or inhibiting the expression of them. Other regulators include endogenous small (19–24 nucleotides) non-coding RNAs (miRNAs) that involve in the regulation of gene expression at the post-transcriptional level [1] by inhibiting the translation procedure or degrading target mRNAs. It has been found that both TFs and miRNAs play an important role in human cancers [1]–[5] by means of controlling many biological processes in cancer development and progression.

Many studies have found that TFs and miRNAs are primary gene regulators in animals and function in a similar regulatory logic [6]. Hence TFs and miRNAs can regulate the same target gene cooperatively at the transcriptional and post-transcriptional level respectively. On the other hand, miRNAs are regulated by TFs during their transcription from the genome within the nucleus and the expression of TFs could also be modulated by miRNAs. Therefore, gene regulations by TFs and miRNAs are often tightly coupled, rendering a particular ‘Feed-forward loops’ (FFLs) [7] structure with closed regulatory circuits. FFLs have been demonstrated as one ofthe most common types of co-transcriptional motifs [8] and it has been reported that hundreds of miRNA-controlled FFLs are available at the genome level [9], [10]. By forming functional modules in gene regulatory networks (GRNs), FFLs control many cell functions and also play an important role in human cancers, for examples, by supporting oncogenic properties of oncogenes [11] and influencing a plenty of target genes in tumor cells by diverse biological pathways [12]. Thus, it becomes crucial to reconstruct and analyze TF–miRNA co-regulatory FFLs in human cancers, in order to find out how miRNAs and TFs cooperate with each other in cancer cells and how they contribute to carcinogenesis.

One of the challenges in studying FFLs is the incomplete information of regulatory targets. As there are only a small number of experimentally verified targets, most FFL studies adopt regulatory information from computational prediction. For example, in order to find cancer-related miRNAs and TFs, Yan *et al*. extracted and ranked FFLs from predicted TF and miRNA targets using TRANSFAC and TargetScan [13]. Ye *et al*. also used FFLs obtained from other prediction resources to construct and analyze miRNA and TF co-regulatory network in T-cell acute lymphoblastic leukemia [14]. However, these predicted results contain a large proportion of false positives, and more critically FFLs generated by above approaches are static and cannot represent the dynamic and conditional regulation relationships under different experimental conditions.

Currently, parallel microarray experiments have been performed to investigate gene and miRNA expression in cancers simultaneously [15], [16], which provides a great opportunity to address aforementioned issues by using expression data in reconstruction of TF–miRNA co-regulatory FFLs. Recently, Lu *et al*. proposed a Lasso regression model that utilizes computationally predicted regulatory interactions and cancer parallel expression data to infer miRNA-target regulatory networks [17], Yu *et al*. used stepwise linear regression model (STEP) that also integrates predicted regulations with expression data to obtain a combinatorial network of TF and miRNA in cancer [18]. These two papers shed light on methodology for building miRNA-involved GRNs and revolutionized our understanding of the implication of TFs and miRNAs in human cancers. However, study of FFLs requires precise co-regulatory information, as FFLs represent themselves as a subtle kind of co-regulatory motif. Therefore more sophisticated computational methods are preferred to accurately reconstruct FFLs from expression data with the aid of predicted regulatory interactions.

In this study, we proposed a novel computational method based on feature selection to reconstruct TF-miRNA co-regulatory FFLs in human cancers. As a powerful machine learning technology, feature selection has been widely used in many areas of bioinformatics, such as gene selection from microarray data [19], inference of gene networks [20], content and signal analysis of sequence [21] and mass spectra analysis [21]. We employed two popular feature selection strategies: filter and wrapper, to efficiently discover co-regulations of TFs and miRNAs from parallel microarray data of human cancers. Our results showed that the proposed method significantly reduced the false discovery rate in the inferred regulatory relationships, leading to more accurate FFL reconstruction. Further analysis of the FFLs identified by the proposed method showed that they included many known cancer-related genes and miRNAs, indicating their functional importance in human cancers.

## Materials and Methods

### Predicted regulatory interactions

Three types of regulatory interactions were investigated in this study: TF to miRNA (TF-miRNA), TF to gene (TF-gene), and miRNA to gene (miRNA-gene). We downloaded predicted TF-miRNA interactions from cGRNB website [18] with 11,599 regulatory pairs. To retrieve candidate TF-gene interactions, TF binding sites were first extracted from the TFbsConsSites file from UCSC [22] by following the procedure described in [18] and then used to scan the 1 kb upstream to 0.5 kb downstream of the transcription start sites of all reference genes in UCSC. The results were further combined to 7,059 TF-gene interactions from TRED database [23], leading to totally 130,338 TF-gene interactions including 16,534 target genes and 214 human TFs. For miRNA-gene interactions, the results of three widely used miRNA target prediction tools: PicTar [24], TargetScan [25] and miRanda [26] were obtained from miRGen database [27], which contain 75,968, 75,613 and 41,804 predicted miRNA-gene interactions respectively. The union of the prediction results, including 118,408 miRNA-gene interactions with 276 human miRNAs and 10,255 target genes, were used for further investigation.

### Parallel mRNA and miRNA expression datasets

We used two parallel mRNA and miRNA expression datasets for human cancers, which represent miRNA and mRNA expression data obtained from the same samples and under the same experiment conditions. The first dataset includes NCI-60 mRNA expression data based on the Affymetrix HG-U133 chips [16] and the parallel miRNA expression data [15] from CellMiner website. This pan-cancer dataset includes totally 60 different human cancer cell lines, originating from melanomas, leukemia and other solid tumors such as breast, colon, ovarian, lung and prostate cancer. This parallel expression dataset consists of totally 8,388 genes and 195 miRNAs [18]. The second parallel mRNA and miRNA expression dataset adopted in this study consists of 111 prostate cancer and 28 normal prostate samples, including 373 miRNAs and 19,253 mRNAs [28]. This dataset is available at the GEO database with accession number: GSE21032.

### Feature selection for identifying regulatory interactions

For each target (mRNA or miRNA), the set of initial features (i.e. all predicted TFs or miRNAs that regulate this target) usually contains more than one element due to a large number of false positives in the predicted results. Then the feature matrix has *n* rows and *m* columns indicating the *n* regulators and their expression values in *m* samples. As the first step, we filtered the feature set of each target using an efficient mRMR method (minimal-redundancy-maximal-relevance) [19] based on mutual information, which is a widely used measure to define dependency of variables. Specifically, let *p*(*x*) and *p*(*y*) be the marginal probability distribution functions of two variables *x* and *y* and *p*(*x,y*) be the joint probability distribution function, the mutual information is defined as: 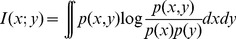
(1)Suppose a target *x* with expression value *E_x_* has two regulators *y_i_* and *y_j_* with expression values *E_yi_* and *E_yj_*. For example, if *y_i_* and *y_j_* represent a miRNA and a TF then *E_yi_* and *E_yj_* will be obtained from the parallel miRNA and mRNA expression data, respectively. The final feature set *S* after the filter step will satisfy two criteria used in the mRMR method, i.e. maximum relevance with the target and the minimal redundancy between regulators (i.e. to select regulators that have not only minimal redundancy in mutual information with respect to existing regulators, but also maximal mutual information with the target), as formulated by equation (2) and (3) respectively. Here *I* represents the mutual information of two variables. From the ranking results of mRMR, we selected up to 20 top candidate regulators that are most likely to have interactions with the target for further investigation, which should include all possible regulators. 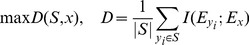
(2)

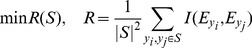
(3)Next, features of each target were further optimized by the wrapper feature selection method, and in this step we employed a recursive backward elimination procedure. We modeled expression values of target *i* (*E_xi_*) and its *p* regulators (*E_yi1_*,…, *E_yip_*) with linear regression (Equation 4), where *β_i_* is the regression coefficient and *ε_i_* represents the error term or noise generated from the regression process. For each time, we deleted the regulator with the smallest regression coefficient from the featureset and repeated the process above with the remaining regulators until the feature set became empty. The optimal features were determined by smallest *p*-value (less than 0.01) of the linear regression model using F-test. 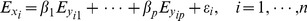
(4)


### Reconstruction and validation of TF-miRNA co-regulatory FFLs

The co-regulatory interactions identified by filter-wrapper feature selection method consist of three kinds of regulatory relationships: TF-gene, miRNA-gene and TF-miRNA. To reconstruct co-regulatory FFLs, we performed an exhaustive search base on depth-first method for all target genes in the results. For TF-FFLs, first a list of potential target genes that are controlled by at least one TF and miRNA were generated. The miRNA regulators of each target gene were then examined one by one and a TF-FFL was generated if the target gene and its miRNA regulator were both controlled by a TF. For miRNA-FFLs, the same target gene list was used and a miRNA regulating both a target gene in the list and its TF was selected to build a miRNA-FFL. Finally, to identify the composite-FFLs the reconstructed miRNA-FFLs were further examined iteratively to see whether the TFs in these FFLs also regulate the corresponding miRNAs.

To validate the FFLs reconstructed from parallel expression dataset and prior information from predicted regulatory interactions, we randomly selected the same number of interactions from predicted regulatory pairs from which we reconstructed the FFLs using above approach. This procedure was repeated 1,000 times to generate the empirical distribution under the null hypothesis that the FFLs reconstructed by our approach indeed arise by chance. We then performed one-sample t-test based on the number of TF-FFLs, miRNA-FFLs and composite-FFLs reconstructed from pan-cancer dataset and calculated the *p*-value to determine whether this null hypothesis can be rejected at significance level of 0.01.

In addition, to further validate the importance of FFLs to cancer, we evaluated the performance of the identified FFLs in classifying prostate cancer and normal samples using parallel gene expression data. LIBSVM [29], a public SVM library, was selected for classification. Leave-one-out cross validation (LOOCV), which is the most objective and rigorous method to assess a classifier, was adopted to evaluate the classification performance of FFLs. For comparision, we also used a baseline method, in which the labels of cancer and normal expression dataset were permuted 100 times. For each time, SVM classifiers using the same FFLs were generated from the permuted expression data and then tested with the same evaluation procedure. The classification results obtained from all permutation tests were averaged to get the baseline performance.

Three classification performance measurements used in this study, accuracy (*Acc*), sensitivity (*Sn*) and specificity (*Sp*) are defined as folllows: 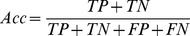
(5)

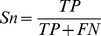
(6)

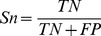
(7)Here *TP*, *TN*, *FP* and *FN* represent true positive, true negative, false positive and false negative, respectively. Meanwhile, since the sizes of the cancer and normal samples are very different, Matthews correlation coefficient (*Mcc*) was used, which is a balanced measurement of the quality of classifications [30]: 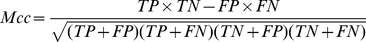
(8)In addition, we also plotted the receiver operator characteristic (ROC) curves for performance comparison, in which the x-axis represents 1- *Sp* and y-axis represents *Sn*.

## Results

### Filter-wrapper feature selection

**Figure 1:**
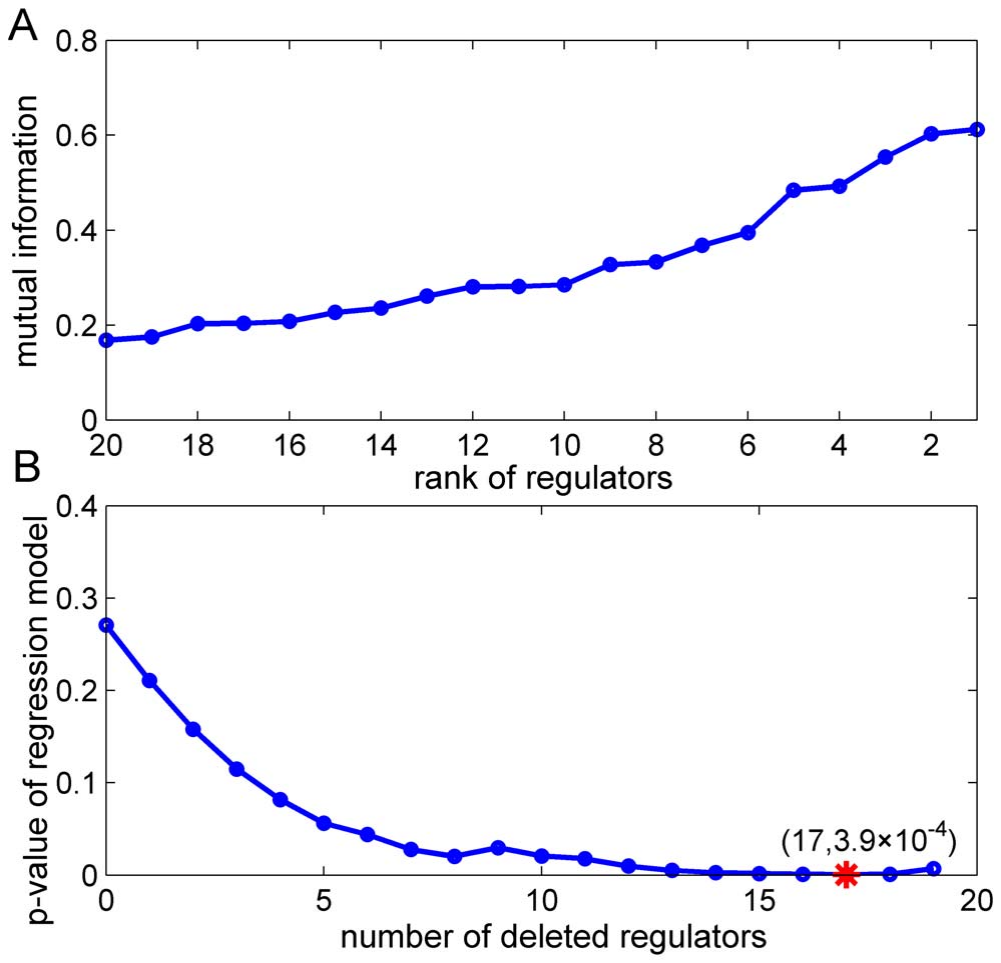
Examples of the filter and wrapper feature selection process. (**A**) An example showing mutual information of all regulators in the filter feature selection process. We chose the top-ranking regulators selected by mRMR, which demonstrate larger mutual information values that indicate high relevance with the target gene. (**B**) An example illustrating *p*-value change of linear regression model in the wrapper feature selection process. When 17 features are removed, the optimal *p*-value (marked by red ‘*’) found by wrapper feature selection is 3.9×10^−4^.

**Figure 2:**
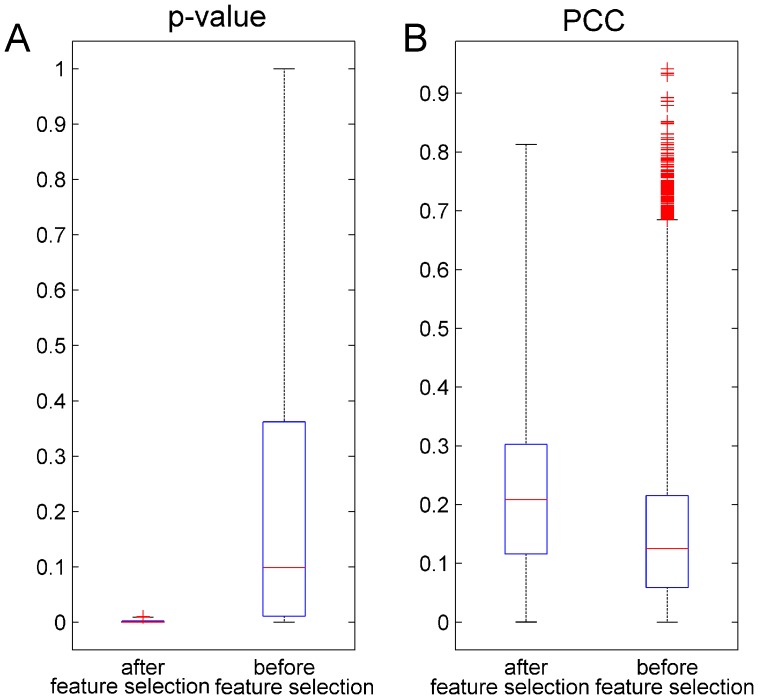
Evaluation of the proposed model. (**A**) *P*-values of the linear regression models for all target genes before and after feature selection. *P*-values significantly decreased after feature selection was performed. (**B**) Pearson correlation coefficients (PCCs) between all target genes and their regulators before and after feature selection. Higher PCCs (U-test *p*-value: 4.4×10^−167^) were observed in the final identified regulatory interactions.

An example of the filter-wrapper feature selection procedure using the pan-cancer dataset is illustrated in Figure 1. In filter feature selection, top-ranking regulators selected by mRMR demonstrate large mutual information, indicating high relevance with the target gene (Figure 1A). Figure 1B shows at the beginning, there are totally 20 candidate features with possible false positives and the *p*-value of associated linear regression model is 0.27. When wrapper feature selection is performed, the corresponding *p*-value dramatically decreases, suggesting a better model that is more close to real gene regulation mechanism. The final model consists of three regulators with an optimal *p*-value of 3.9×10^−4^. Furthermore, the boxplot of the *p*-values for all target investigated in this study (Figure 2A) shows feature selection generally renders more accurate regression models. We also evaluated the proposed method by calculating the Pearson correlation coefficient (PCC), a widely used measurement for identifying regulatory relationships [31], between candidate regulators and target. As a result, significantly higher PCCs (U-test *p*-value: 4.4×10^−167^) were observed in the regulatory interactions after feature selection (Figure 2B). Taken together, the filter-wrapper feature selection method greatly improves the reliability of identified regulatory interactions by accurately modeling parallel expression data and efficiently removing falsely predicted interactions.

To further assess the performance of the proposed method, we permuted the expression values of expression data randomly for 100 times and calculated false discoveryrate (FDR) by regarding interactions generated from the randomized datasets as false positives. For comparison, we also evaluated the performance of two other methods: Lasso [17] and STEP [18]. The testing process was repeated 3 times and the results are shown in Table 1. With comparable variance, the mean value of FDR for filter feature selection is 0.11, which is significantly smaller than that of Lasso and STEP. Furthermore, by using filter and wrapper feature selection together, we observed a dramatically reduction in FDR variance from 1.24 to 0.38, indicating better consistency and robustness compared to other approaches. Furthermore, this method yielded a mean FDR of 0.06, which is 5% better than the filter method. Taken together, these results demonstrate the superior performance of filter-wrapper feature selection method.

**Table 1:** Comparison of false discovery rate for different methods.

Method	STEP	Lasso	Filter feature selection	Filter-wrapper feature selection
FDR mean	0.33	0.23	0.11	**0.06**
FDR variance	1.93	1.23	1.17	**0.38**

Finally, we investigated miRNA regulatory interactions before and after feature selection. As shown in Table 2, there were totally 190,976 predicted miRNA-target interactions and most of them were removed when feature selection was applied, indicating these predicted regulatory interactions were either false positives or unrelated to human cancers. In addition, by resorting to miRTarBase [32] that contains experimentally validated miRNA targets, we found the fraction of known regulatory interactions was significantly increased in the results (Table 2, Hypergeometric-test, *p*-value: 2.4×10^−3^ for filter feature selection, 4.0×10^−4^ for filter-wrapper feature selection), which also supports the utility of feature selection in discovering regulatory interactions.

**Table 2:** Statistics of miRNA-target regulatory interactions before and after feature selection.

miRNA-target interactions	Before feature selection	Filter feature selection	Filter-wrapper feature selection
Total number	190976	4108	1696
Fraction of known interactions (%)	0.4	0.7	1.0
Hypergeometric-test *p*-value		2.4×10^−3^	4.0×10^−4^

### TF and miRNA co-regulatory FFLs in human cancers

**Figure 3:**
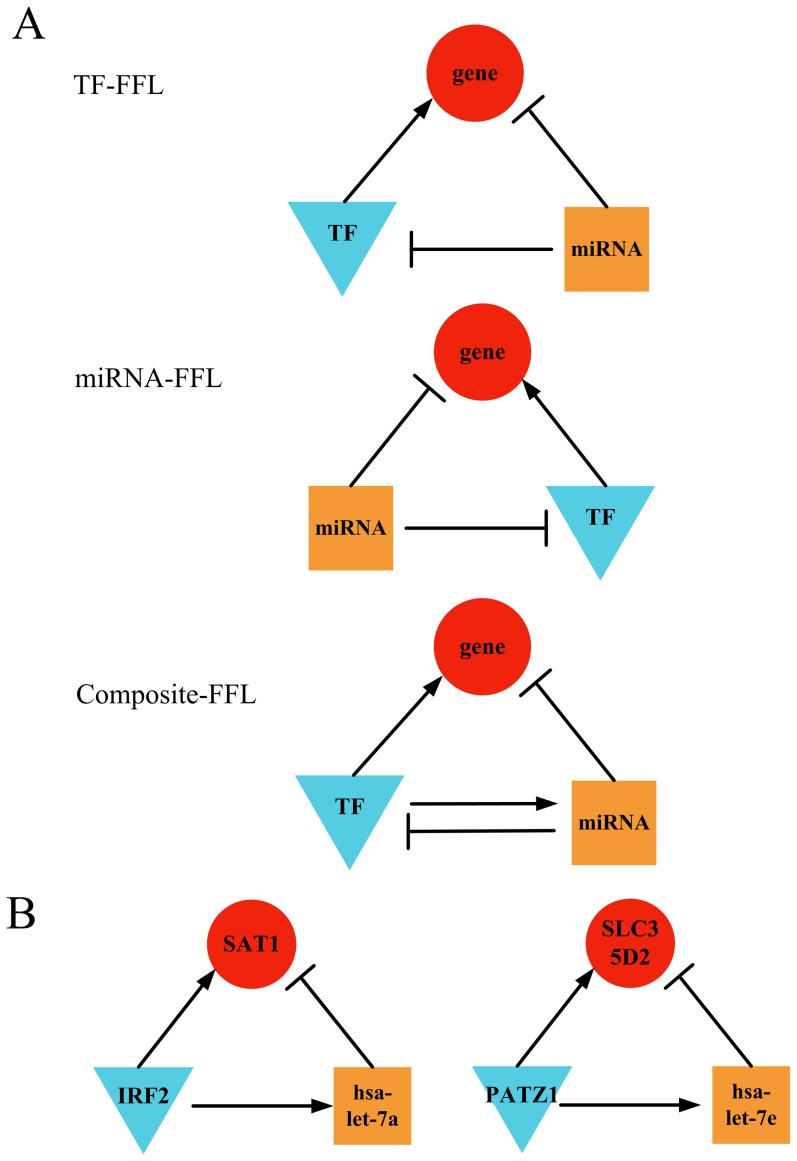
Co-regulated FFLsin human cancers. (**A**) Three types of 3-vertex FFLs found in human cancers. According to the relationship between the miRNA and TF, the mixed FFLs found in human cancers were classified as the TF-FFL (the TF directly regulates the miRNA and target gene while the miRNA also regulates the target gene), miRNA-FFL (the miRNA directly regulates the TF and target gene while the TF also regulates the target gene) or composite-FFL (the TF and the miRNA regulate each other while they also regulate the same target gene). (**B**) Common TF-FFLs found in both pan-cancer and prostate cancer datasets. Red circles indicate target genes; blue triangles and orange squares indicate TFs and miRNAs.

From the 24,033 regulatory interactions identified by feature selection, we identified three types of FFLs in human cancers (Figure 3A): TF-FFL, miRNA-FFL and composite-FFL. In the TF-FFL, the TF is the main regulator that directly regulates the miRNA and target gene while the miRNA also regulates the target gene. The miRNA-FFL has the same structure with TF-FFL but the main regulator is instead miRNA. The composite-FFL is a combination of TF-FFL and miRNA-FFL, in which the TF and the miRNA regulate each other while they also regulate the same target gene. Note these FFLs have also been reported in other studies of cancer [7], [13], [14], [33], [34], suggesting the prevalence of FFLs in gene regulation and mechanism of carcinogenesis. From the pan-cancer dataset, we successfully reconstructed 98 TF-FFLs, 106 miRNA-FFLs and 4 composite FFLs from the identified regulatory interactions by feature selection. To further validate these cancer-related FFLs, we performed one-sample t-test by comparing reconstructed FFLs to those generated by randomly selected interactions from predicted regulatory pairs (see Method), and the average number of TF-FFLs, miRNA-FFLs and composite FFLs in randomized interactions was 56, 26 and 0.2, which were all significantly lower (*p*-value: 1.2×10^−8^ for TF-FFL, 2.0×10^−43^ for miRNA-FFL, 9.9×10^−11^ for composite-FFL) than the number of FFLs identified in human cancers.

Meanwhile, we reconstructed TF-miRNA co-regulatory FFLs using the prostate cancer dataset and compared them with those generated from normal prostate expression data. The number of co-regulatory FFLs in prostate cancer was 110, and much more FFLs (425 in total) were identified in normal prostate cells. Moreover, we found the composition of FFLs was also significantly different (chi-square test, *p*-value: 1.7×10^−2^), for example, the percentage of miRNA-FFLs was 79% in prostate cancer while this number increased to 89% in normal prostate tissue. This phenomenon implies many normal co-regulatory FFLs are dramatically suppressed or altered in prostate cancer. Finally, we compared the FFLs reconstructed from pan-cancer and prostate cancer datasets and identified 2 TF-FFLs (Figure 3B) that appeared in both datasets. Interestingly, the miRNAs in these two FFLs, hsa-let-7a and hsa-let-7e, belong to the hsa-let-7 family that is related to prostate [35], breast [36], lung [37] cancers.

**Figure 4:**
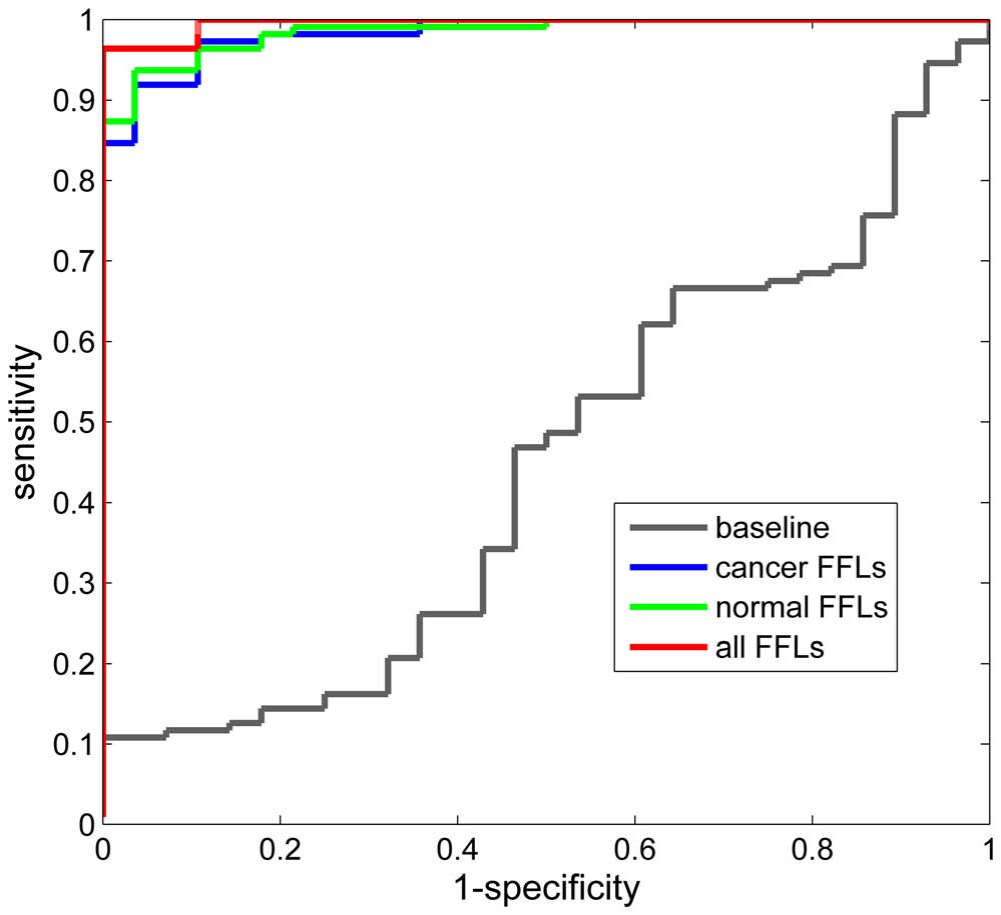
The receiver operator characteristic (ROC) curves of classification approches using different identfied FFLs. The gray, blue, green and red curves are the ROC curves of baseline (one permutation), cancer FFLs, normal FFLs and all FFLs, respectively. The largest area under the curve indicates the best performances of classification.

In addition, we performed classification analysis of prostate cancer and normal samples by using the expression data of miRNAs and genes in aforementioned co-regulatory FFLs. The performance of normal and cancer FFLs were evaluated by using LOOCV and shown in Table 3. Both normal and cancer FFLs yield very good classification results with *Acc* of 95.0% and 95.7%, repectively. Also the *Sn*, *Sp* and *Mcc* measurements show the classfication results of both kinds of FFLs are very balanced. In addition, by using all these FFLs the classification performance is further improved with *Acc*, *Sn* and *Mcc* increasing to 97.1%, 99.1% and 0.909, respectively, which are signficantly better than those of the baseline method. These results are also corroborated by the ROC curves of above approaches (Figure 4), suggesting the effectiveness of these FFLs in classifying prostate cancer and normal samples and their importance to cancer.

**Table 3:** Comparison of classification performance using identified FFLs.

Data	ACC	SN	SP	MCC
Baseline	71.2%	86.5%	10.7%	−0.033
Normal prostate FFLs	95.0%	96.4%	89.3%	0.846
Prostate cancer FFLs	95.7%	97.3%	89.3%	0.866
All FFLs	97.1%	99.1%	89.3%	0.909

### Key players in cancer-related FFLs

We calculated the occurrence of the TFs and miRNAs in pan-cancer and prostate cancer FFLs. As shown in Table 4, the top ranked TF and miRNA are STAT3 and hsa-let-7e, respectively. Interestingly, we found STAT3 appeared in 18 FFLs of prostate cancer (Table 5), which was significantly enriched compared to those in normal prostate tissue (chi-square test, *p*-value = 2.6×10^−12^) and in pan-cancer (chi-square test, *p*-value = 3.8×10^−3^). This phenomenon implies STAT3 is implicated in prostate cancer. It has been reported that STAT3 is constitutively activated in prostate cancer tissue [38] and induction of STAT3 expression can induce a malignant change of normal prostate epithelial cells [39]. Furthermore, STAT3 has been shown as a promising therapeutic target for prostate cancer [40]. All these observations demonstrate that STAT3 is an important cancer-related TF and has a prominent impact on occurrence and development of prostate cancer. Moreover, we discovered more than 24% of the target of STAT3 found in pan-cancer also appeared in prostate cancer. Further analysis of functional enrichment of STAT3 targets in prostate cancer showed these target played an important role in various cellular process regulations implicated in carcinogenesis, for example, cell surface binding and the activity regulation of different kinds of proteinases (Table 6). The results of pathway analysis also indicated these target were abundant in Fructose and mannose metabolism and Notch signaling pathway.

**Table 4:** Occurrence of the TFs and miRNAs in pan-cancer and prostate cancer FFLs.

	Prostate cancer		Pan-cancer		
TFs					
Gene	FFLs	Rank	FFLs	Rank	Total FFLs
STAT3	18	1	13	6	31
HAND1	9	3	14	4	23
E2F2	7	4	7	10	14
MYB	2	17	12	7	14
PATZ1	4	10	8	9	12
SRF	7	5	4	22	11
CUX1	6	6	1	32	7
NFYC	4	9	2	27	6
MYOD1	1	25	4	21	5
RREB1	1	26	3	24	4
IRF2	2	16	1	34	3
HSF1	1	23	1	33	2
miRNAs					
hsa-let-7e	41	1	68	1	109
hsa-let-7b	19	2	18	4	37
hsa-let-7c	13	5	24	2	37
hsa-let-7a	16	3	18	3	34
hsa-let-7i	16	4	12	6	28
hsa-let-7d	2	6	15	5	17
hsa-let-7g	1	8	9	7	10
hsa-let-7f	2	7	8	11	10

**Table 5:** Statistics of STAT3-involved FFLs.

Data	STAT3 FFLs	Total FFLs	*P*-value
Prostate cancer	18	110	
Pan-cancer	13	208	3.8×10^−3^
Normal prostate	5	425	2.6×10^−12^

**Table 6:** Functional enrichment analysis of STAT3 targets in prostate cancer.

Category	Term	Count	*P*-value
GOTERM_MF_FAT	GO:0004857∼enzyme inhibitor activity	13	0.011
GOTERM_MF_FAT	GO:0004866∼endopeptidase inhibitor activity	8	0.033
GOTERM_MF_FAT	GO:0030414∼peptidase inhibitor activity	8	0.042
GOTERM_MF_FAT	GO:0043499∼eukaryotic cell surface binding	3	0.048
KEGG_PATHWAY	hsa00051:Fructose and mannose metabolism	5	0.008
KEGG_PATHWAY	hsa04330:Notch signaling pathway	5	0.024

### TF-miRNA co-regulatory networks in human cancers

**Figure 5:**
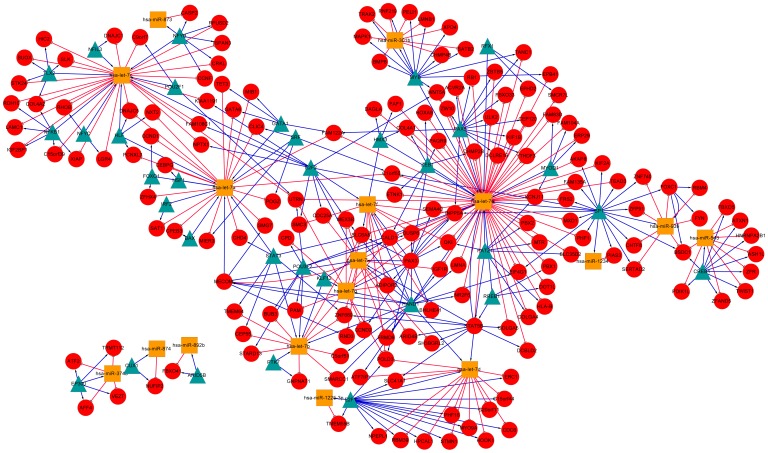
TF-miRNA co-regulatory network based on FFLs reconstructed from the pan-cancer dataset. Red circles indicate target genes; blue triangles and orange squares indicate TFs and miRNAs. Red T shape edge: miRNA regulation; blue arrow shape edge: TF regulation.

**Figure 6:**
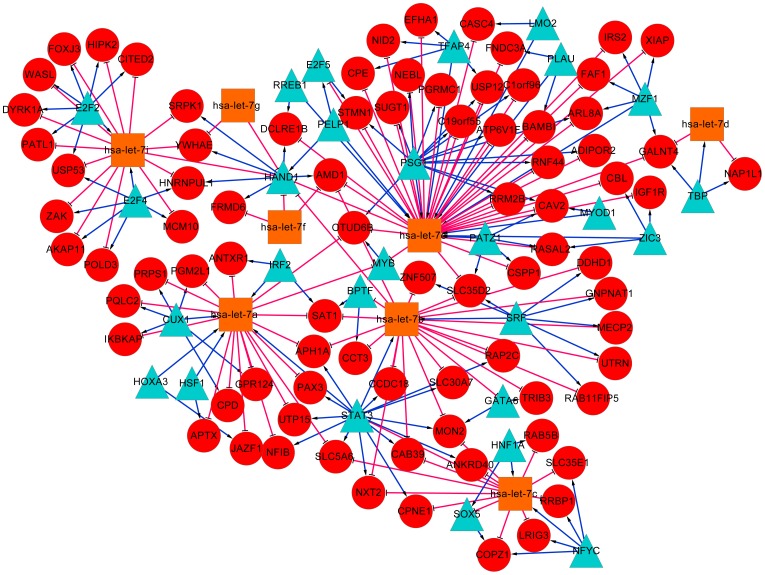
TF-miRNA co-regulatory network based on FFLs reconstructed from the prostate cancer dataset. Red circles indicate target genes; blue triangles and orange squares indicate TFs and miRNAs. Red T shape edge: miRNA regulation; blue arrow shape edge: TF regulation.

Based on the FFLs reconstructed in the two datasets, we further built pan-cancer and prostate cancer specific TF-miRNA co-regulatory networks, and visualized them by using Cytoscape software [41]. As shown in Figure 5, the pan-cancer co-regulatory network contains a total number of 213 nodes, including 37 TFs, 17 miRNAs and 159 other genes. The prostate cancer specific network consists of 118 nodes with 27 TFs, 8 miRNAs and 83 other genes (Figure 6). We calculated the degree (connectivity) of each node and found that the hub with the largest degree in both networks was the same miRNA hsa-let-7e. This result agreed with the FFLs analysis discussed above, indicating that hsa-let-7e may be crucial in various human cancers. Further literature study shows that hsa-let-7e is a member of let-7 family that emerged as tumor suppressor [36] and has been reported to play an important role in the regulation of oncogenes in multipletumors [42], [43].

**Figure 7:**
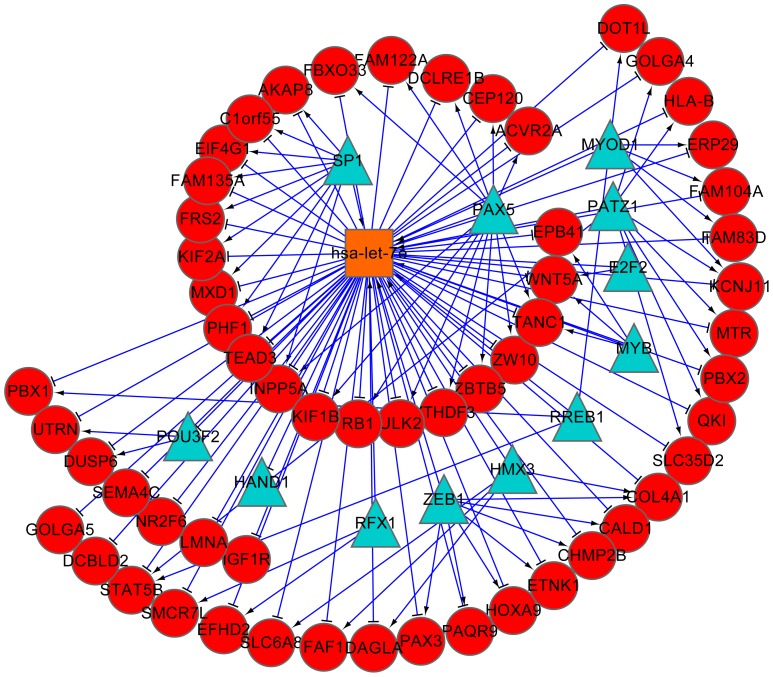
Subnetwork of miRNA hsa-let-7e base on FFLs found in pan-cancer dataset. The subnetwork was drawn with all direct linked nodes of hsa-let-7e, which is shown to be the hub of the co-regulatory network.

The subnetwork of hsa-let-7e retrieved from pan-cancer FFLs (Figure 7) includes 57 target genes and 12 TFs including many cancer-related genes such as MYB, E2F2 and HAND1. MYB is proto-oncogene that has been identified to cause a range of leukemia [44]. E2F2 is cell-cycle regulator whose expression level increases in the prostate cancer tissue [45]. HAND1 has also been reported to play a critical role in carcinogenesis process [46]. We also conducted functional enrichment analysis of hsa-let-7e targets in pan-cancer and the results in Table 7 show that they are significantly enriched in five pathways (hsa05200: Pathways in cancer; hsa05220: Chronic myeloid leukemia; hsa00270: Cysteine and methionine metabolism; hsa05222: Small cell lung cancer; hsa05219: Bladder cancer), among which four pathways are related to human cancers. In all, these results show that hsa-let-7e can inhibit the process of tumor occurrence and development in different tumors by regulating different oncogenes.

**Table 7:** Functional enrichment analysis of hsa-let-7e targets in pan-cancer.

Category	Term	Count	*P*-value
GOTERM_MF_FAT	GO:0003700∼transcription factor activity	23	3.8×10^−5^
GOTERM_MF_FAT	GO:0043565∼sequence-specific DNA binding	17	8.6×10^−5^
GOTERM_MF_FAT	GO:0030528∼transcription regulator activity	27	6.1×10^−4^
GOTERM_MF_FAT	GO:0003677∼DNA binding	36	7.4×10^−4^
GOTERM_MF_FAT	GO:0016564∼transcription repressor activity	10	0.001
GOTERM_MF_FAT	GO:0016831∼carboxy-lyase activity	3	0.027
GOTERM_MF_FAT	GO:0003777∼microtubule motor activity	4	0.030
GOTERM_MF_FAT	GO:0003774∼motor activity	5	0.037
GOTERM_MF_FAT	GO:0008134∼transcription factor binding	10	0.038
GOTERM_MF_FAT	GO:0004652∼polynucleotide adenylyltransferase activity	2	0.043
KEGG_PATHWAY	hsa05200:Pathways in cancer	8	0.026
KEGG_PATHWAY	hsa05220:Chronic myeloid leukemia	4	0.029
KEGG_PATHWAY	hsa00270:Cysteine and methionine metabolism	3	0.037
KEGG_PATHWAY	hsa05222:Small cell lung cancer	4	0.039
KEGG_PATHWAY	hsa05219:Bladder cancer	3	0.054

## Discussion

Human cancers are usually characterized by proliferation of versatile genes at different stages of development with complicated regulatory mechanism, therefore reconstruction of gene regulation in human cancers, especially with respect to its complex, dynamic and conditional feature, can greatly advance our knowledge on the origin of cancer and its malignant behavior. In this study, we utilized filter-wrapper feature selection method to identify regulatory interactions between target genes and regulators, from which we further reconstructed TF-miRNA co-regulatory FFLs in human cancers. The proposed method takes full advantage of parallel expression datasets and prior information from predicted regulatory interactions to model and characterize the complicated co-regulation mechanism in human cancers. By this way, we successfully filtered out a large proportion of false positives in the predicted regulatory interactions and obtained more accurate miRNA-TF co-regulatory FFLs by greatly reducing the false discovery rate. The results show that by combining information from TF binding site and seed–pairing of miRNA with the dynamic expression of gene and miRNA in human cancers, we can efficiently infer the complicated co-regulation mechanism of miRNAs and TFs under different experimental conditions.

Recent studies [13], [14] have shown that regulations by miRNAs and TFs are tightly coupled in FFLs, which inspired us to concentrate on the regulatory network based on FFLs that may work as the ‘core’ of the whole gene regulatory network. In the key player analysis, we found two important regulators STAT3 and hsa-let-7e in human cancers. We also identified hsa-let-7e as the hub of both networks in the analysis of the pan-cancer and prostate cancer co-regulatory networks. Further topological analysis shows that both degree distributions of the two networks approximate power law functions with the degree exponents smaller than 3, which indicate that both networks exhibit distinct topological properties such as small-world, scale-free, nonrandom and non-uniform [47], rendering high robustness against accidental node failures.

At the same time, we also found some of the results reported in this study are consistent with the results found in other studies of cancer FFLs. For example, in accordance with the conclusion that has-let-7e is key regulator in human cancer, in the work of Ye *et al*. [14], has-let-7e is also found to be related to T-cell acute lymphoblastic leukemia. Also, we reported that MYB is a top-ranked TF in pan-cancer and prostate cancer, which is also verified by the work of Yan *et al*. [13] that lists MYB as one of the most frequently appeared TFs in FFLs across six cancer datasets including prostate cancer. Moreover, in this study three cancer related genes, E2F1, SP1 and STAT3, are found to form multiple significant FFLs, which are also corroborated by the results in [13]. The similarity of these findings suggests the importance of these miRNAs/genes in cancer and the reliability of the method proposed in this study.

While our approach has demonstrated its utility in reconstruction of co-regulatory FFLs, it is limited to studies where the expression of both gene and miRNA are experimentally determined. However it is expected that more parallel miRNA and mRNA expression datasets will be available given the fact that many related researches are now underway or will be conducted in the near future. In addition, high-throughput technologies, such as microarrays and next-generation sequencing are becoming cost-efficient and therefore affordable in caner studies with a large number of patients involved. On the other hand, the FFLs identified in this work can be further refined if knowledge of human cancers can be applied in the modeling procedure, especially information regarding cancer-related miRNAs and TFs that have verified functional roles and altered expression in human cancers. Finally, in this work we focused on 3-vertex FFLs since they are the most prevalent and representative [7], [13], [14]. In the future work we will take other types of FFLs, such as FFLs with 4 vertexes [7], into account and analyze them in more comprehensive ways, for example, by exploring the causal regulatory relationships of miRNA-gene and TF-gene [19], or modeling the difference of regulation mechanisms of TFs and miRNAs to specific target genes.

## Conclusions

To investigate the co-regulatory mechanism in human cancers, we proposed anovel filter-wrapper feature selection method to accurately identify dynamic and conditional regulation relationships by integrating prior regulation information with parallel expression data. Our results showed the proposed method achieved better performance with much lower false discovery rate than existing approaches. By applying this method to two human cancer datasets, we successfully reconstructed co-regulatory FFLs mediated by TFs and miRNAs. Moreover, the indentified FFLs showed effectiveness in classifying prostate cancer and normal samples. Further analysis showed that the top-ranking regulator STAT3 was significantly enriched in cancer-related FFLs, suggesting its crucial regulatory role in human cancers. Meanwhile, another top-ranking regulator, miRNA hsa-let-7e, was shown to be the hub of the two FFL-based co-regulatory networks, which highlights its functional importance as a tumor suppressor. In addition, functional enrichment analysis demonstrated that the targets of these two key regulators in cancer-related FFLs were significantly enriched in cellular process regulations implicated in carcinogenesis and cancer-related signaling pathways. In conclusion, we introduced an efficient computational approach to reconstruct co-regulatory FFLs by identifying gene regulatory interactions with feature selection, which can be generally applied to other gene regulation studies using parallel expression data with respect to different biological contexts.
